# Clinicopathologic features of endometrial cancer in Chinese patients younger than 50 years with a family history of cancer

**DOI:** 10.1097/MD.0000000000012968

**Published:** 2018-10-26

**Authors:** Yuan He, Xiang Tao, Feifei Huang, Nan Jia, Yan Du, Jinming Yu, Weiwei Feng

**Affiliations:** a School of Public Health, Fudan University; b Obstetrics and Gynecology Hospital of Fudan University; c Ruijin Hospital, Shanghai Jiaotong University School of Medicine, Shanghai, China.

**Keywords:** clinicopathologic features, endometrial cancer, family history of cancer, younger than 50 years

## Abstract

Genetic factors play an important role in shaping the biologic characteristics of malignant tumors, especially in young patients. We aimed to determine the clinicopathologic features of endometrial cancer (EC) in patients younger than 50 years with a family history of cancer.

Overall, 229 patients with EC, including 40 with a positive family history of cancer (PFH) and 189 with a negative family history of cancer (NFH), were enrolled in this case–control study. The family history of cancer in a 2-generation pedigree was recorded for the PFH group. Clinicopathologic features such as menarche age, body mass index, personal cancer history, grade, and histologic type were compared between the 2 groups. Mismatch repair (MMR) proteins including MLH1, PMS2, MSH2, and MSH6 were assessed by immunohistochemistry (IHC) in surgical samples. Univariate (Pearson Chi-squared test, Fisher exact test, *T* test, Wilcoxon rank sum test, logistic regression) statistics and stepwise multivariate logistic regression were used to identify factors associated with PFH in the analysis.

Among young patients with EC, the PFH group had younger age-of-onset age of endometrial cancer (≤40 years) (odds ratio [OR] = 2.21, 95% confidence interval [95% CI]: 1.01–4.82) than the NFH group. The proportion of overweight/obese patients was high in both the NFH (58.7%) and PFH (80%) groups. Colorectal, lung, endometrial, breast, and hepatocellular carcinoma accounted for 58.6% of all cancer types among 1st- and 2nd-degree relatives. Additionally, 19.2% of patients displayed deficiency in at least 1 MMR protein, with a significantly higher proportion of MMR protein deficiency in the PFH group than in the NFH group (adjusted OR = 4.81, 95% CI: 2.14–8.83).

Clinicopathologic features differ for young patients with EC with and without a family history of cancer. Surveillance of age-of-onset and family history of endometrial cancer, reduction of barriers to healthy lifestyles, and development of risk-appropriate Lynch syndrome screening tools, such as IHC, are needed for these women in Shanghai and other developing cities in China.

## 1 Introduction

Endometrial cancer (EC) is the 5th most common cancer in women, and nearly 11.7% of the cases occur in China.^[1,2]^ The incidence of EC has steadily increased in the past 10 years in China; the age-standardized incidence rates increased from 3.9/10^5^ in 2003 to 5.6/10^5^ in 2007 based on 32 Chinese cancer registries, and the incidence in some areas, such as Shanghai, increased to as high as 14.7/10^5^ in 2013.^[3,4]^ In developed areas of China, EC has supplanted cervical cancer and ranks 1st among gynecologic malignancies. An estimated 63,400 new cases of uterine corpus cancer (the majority of cases were EC) occurred in 2015, with an annual percentage change in the incidence rate of 3.7% during 2000 to 2011 from a study involving 72 Chinese cancer registries.^[5]^ EC occurs primarily in postmenopausal women older than 60 years. However, during the past decade in China, EC incidence has rapidly increased for women 30 to 35 years of age, and nearly 10% to 15% of EC cases occurred in women aged 50 years or younger.^[6,7]^ Young women with EC have thus posed a challenge to gynecologic oncologists and public health experts in China.

The EC in young women with a positive family history of cancer or personal history of synchronous/metachronous cancer can be indicative of Lynch syndrome (LS), which is caused by germline mutations in the mismatch repair (MMR) genes MLH1, MSH2, MSH6, and PMS2.^[8–10]^ Current criteria, such as the Amsterdam II and revised Bethesda guidelines, depend on analyzing a detailed family history of cancer.^[11,12]^ From a clinical standpoint, a family history can be a very powerful risk assessment tool and can either significantly increase or decrease the concern for LS.^[13]^ In China, whether young women with EC with a positive family history of cancer have unique clinicopathologic and MMR protein features relative to women with no family history is not well studied. Studies focused on women with EC and a family history of cancer are mostly based on Caucasian populations and can be classified into the following types: estimating the association between family history and risk of EC^[14,15]^; screening the EC cases for LS risk by gathering family history data to compare with immunohistochemical (IHC) testing, microsatellite instability testing, or genetic testing results.^[8,16,17]^ Few studies have reported the clinicopathologic features and universal IHC screening of Chinese women aged 50 years or younger. Considering the ethnic differences in patients with EC, to provide clues for interventions in young women with EC as well as for the prevention of EC in family members, this hospital-based case–control study was designed in the largest Obstetrics and Gynecology Hospital in China. Investigation and assessment of a 2-generation pedigree and a universal screening with immunohistochemistry were conducted, and data on the reproductive health and pathologic features of patients were obtained to compare the clinicopathologic features of patients with or without a family history of cancer.

## 2 Materials and methods

### 2.1 Setting and population

**Figure 1:**
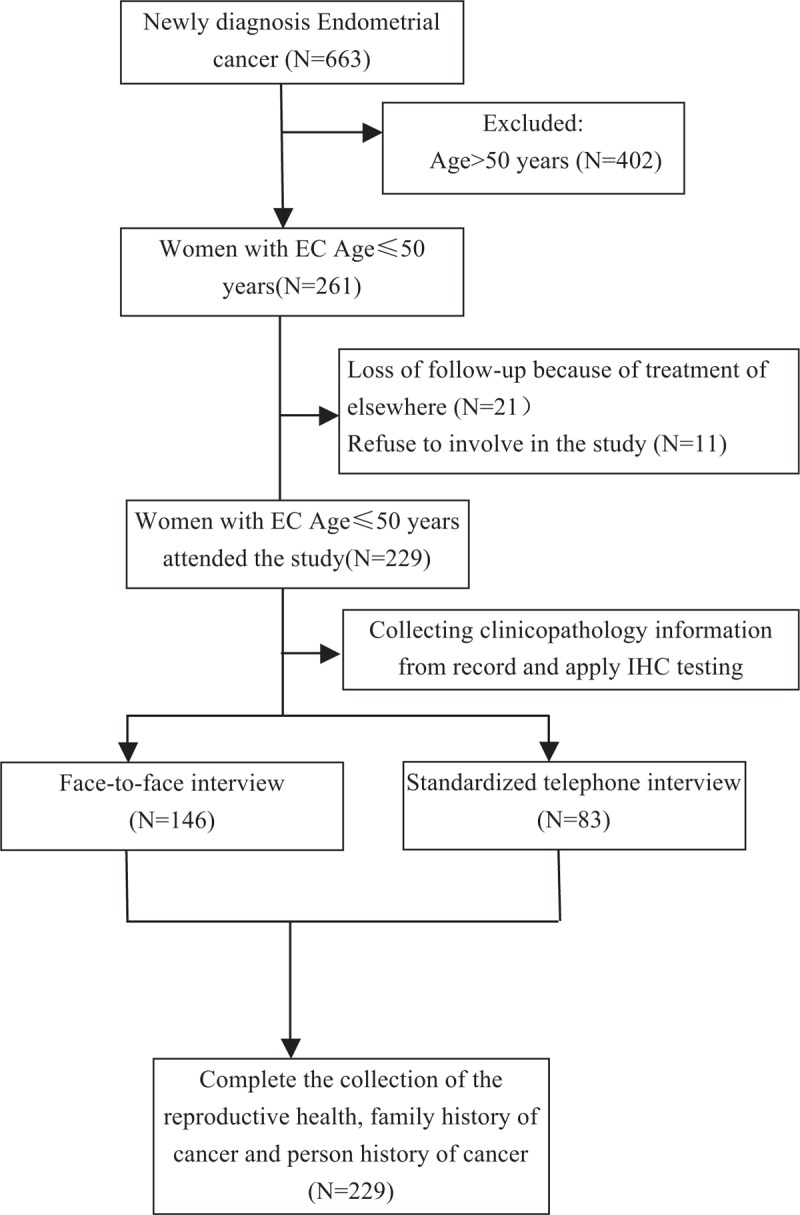
The flowchart of the participants.

This case–control study was approved by the Ethics Committee of Obstetrics and Gynecology Hospital of Fudan University. Data from patients with newly diagnosed EC between November 1, 2014, and October 31, 2016, were reviewed. Eligibility criteria included the following: patients undergoing a surgical hysterectomy, patients with histologically confirmed EC of any histologic subtype (including endometrioid, serous, mucinous mixed, clear cell carcinoma, and so on) and any International Federation of Gynecology and Obstetrics (FIGO) (2014) stage, and patients aged 50 years or younger. Informed consent was obtained from all eligible subjects. Of the 261 individuals who fulfilled the selection criteria, 21 individuals were lost to follow-up because of treatment elsewhere, and 11 individuals refused to participate in the study. Finally, a total of 229 patients with EC were included in the study (Fig. 1). The individuals were classified into a case or control group based on their family history of cancer [40 women with a positive family history of cancer (PFH) in the case group and 189 women with a negative family history of cancer (NFH) in the control group].

### 2.2 Medical records

The demographic and clinical information of patients, including age at the time of diagnosis, body mass index (BMI), complications (such as diabetes and hypertension), histologic features (such as the histologic type, grade, status of myometrial invasion, lymph node metastasis, and international FIGO stage), was collected from medical records.

### 2.3 Questionnaire

All individuals were interviewed during their hospital stay or 1 to 3 months after surgery using a structured questionnaire. The questionnaire consisted of the following 3 sections and a total of 15 items developed by a group of 5 gynecologic oncology experts: (I) Reproductive history, including the history of abortion, gravidity, parity, contraception, and menarche. (II) Personal history of cancer, including the type of cancer (except EC) and the age of onset. (III) Family history of cancer in 1st-degree relatives (FDRs; parent, sibling, child of the patient) and 2nd-degree relatives (SDRs; maternal or paternal grandmother or grandfather or aunt or uncle, etc.). For each patient with EC, a specifically trained research investigator took approximately 10 to 15 minutes to complete a face-to-face interview in an outpatient clinic/ward to collect data on the family history of cancer with a detailed 2-generation pedigree. If the detailed information was not obtained, a standardized telephone interview was conducted by trained research nurses to collect a detailed family history of cancer as a complementary method to the face-to-face interview (83 individuals had telephone interviews). Relatives were interviewed for confirmation of the family history of cancer. Questionnaire-aided interviews and medical records review occurred independently.

### 2.4 Immunohistochemistry

A universal IHC screening was performed for MMR proteins in subjects. All IHC analysis results were interpreted by specialized gynecologic pathologists.

**Figure 2:**
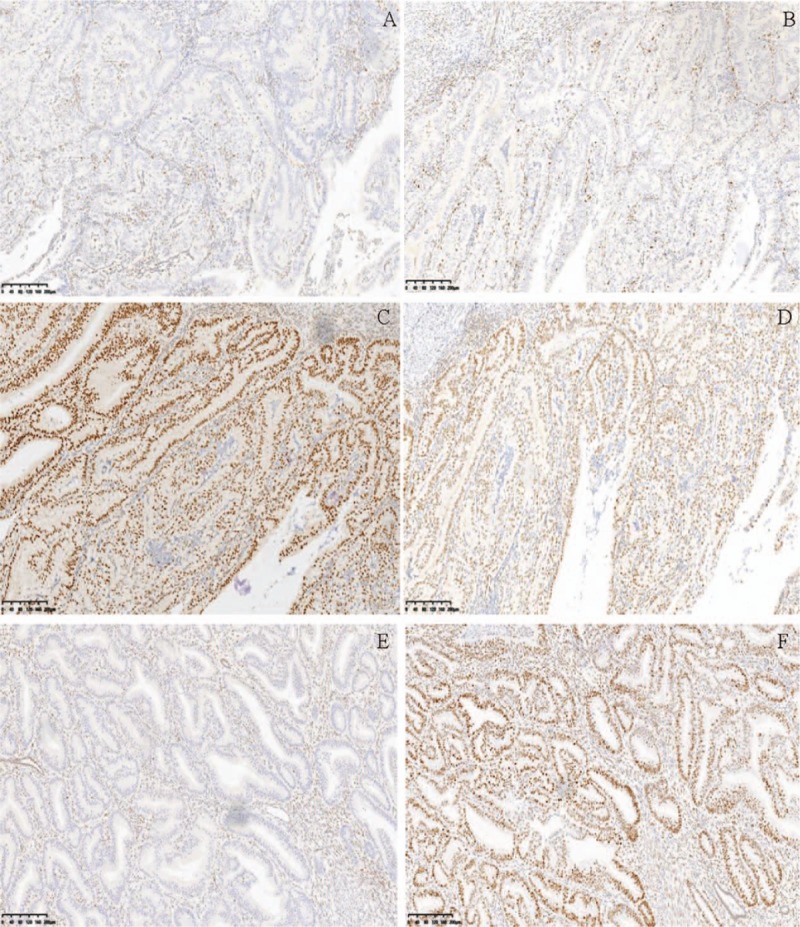
Abnormal mismatch repair protein immunohistochemistry. Two endometrial cancer cases, the 1st showing loss of MSH2 expression (A) and loss of MSH6 (B) in the tumor cell nuclei, compared with the positively staining adjacent stromal cell (yellowish-brown) and retention of expression of MLH1 (C) and PMS2 (D). The 2nd endometrial cancer case displays loss of MLH1 expression (E), compared with retention of expression of MSH6 (F). All photomicrographs taken at magnification: ×200.

The IHC staining of MMR proteins such as MLH1, MSH2, MSH6, and PMS2 was carried out on histologic EC sections. Testing was performed with a Leica Bond Max detection system using the following monoclonal antibodies: MLH1 (DAKO-ES05), PMS2 (DAKO-EPS1), MSH2 (DAKO-FE11), and MSH6 (DAKO-EP49). Nuclear labeling of MLH1, PMS2, MSH2, and MSH6 in the presence of an internal positive control of normal lymphocytes and/or stromal cells was considered positive staining. Negative expression of MLH1, PMS2, MSH2, or MSH6 staining in epithelial cancer cells was considered deficient, while positivity of at least a portion of the cancer cells (more than 5%) was considered intact expression (Fig. 2).

### 2.5 Statistical analysis

Continuous variables were reported as mean ± standard deviation if normally distributed and as median (interquartile range) if non-normally distributed. Demographics, reproductive health, clinicopathology characteristics, and MMR protein expression between the NFH and PFH groups were compared. Two sample Student *t* tests were used for normally distributed continuous variables, Wilcoxon rank sum tests for non-normally distributed continuous variables, Pearson Chi-squared tests or Fisher exact tests for categorical variables. BMI was classified according to World Health Organization Asia-Pacific criteria.^[18]^ Variance inflation factors (VIFs) were used to assess multicollinearity, and a VIF >4 was considered evidence of multicollinearity. Crude odds ratios (ORs) using maximum likelihood estimates were estimated by univariate logistic regression models. A multivariate stepwise logistic regression was performed for adjusted ORs. Variables in the stepwise multivariate logistic analysis included age-of-onset of endometrial cancer, BMI, age of menarche, personal history of cancer, FIGO stage, cervical involvement and the expression of MMR protein. A *P*-value <.05 was considered statistically significant. All analyses were performed using SAS software using SAS 9.4 version (SAS Institute, Inc, Cary, NC).

### 2.6 Ethical approval

This study was approved by the Institutional Ethics Committee of the Obstetrics and Gynecology Hospital of Fudan University.

## 3 Results

Forty patients in the PFH group reported 60 FDRs or SDRs with cancer (Table 1). Twenty-six relatives were from the families in which the proband had a deficient MMR protein expression (proMMR−), and 34 relatives were from the families in which the proband had a positive MMR protein expression (proMMR+). Lung (26.5%), breast (14.7%), and hepatocellular (11.8%) carcinoma were the most common cancer types in relatives from proMMR+ families, while colorectal cancer (50%) was the top cancer type in relatives from proMMR− families. The proportion of family history of cancer was higher in proMMR− families (21/26, 80.7%) than in FDRs in proMMR+ families (23/34, 67.6%). Thirteen relatives (50%) in proMMR− families were diagnosed with colorectal cancer, and the rate was approximately 2.9% in proMMR+ families (*P* < .05). No significant differences were found in the distribution of EC between the proMMR+ and proMMR− families.

**Table 1:**
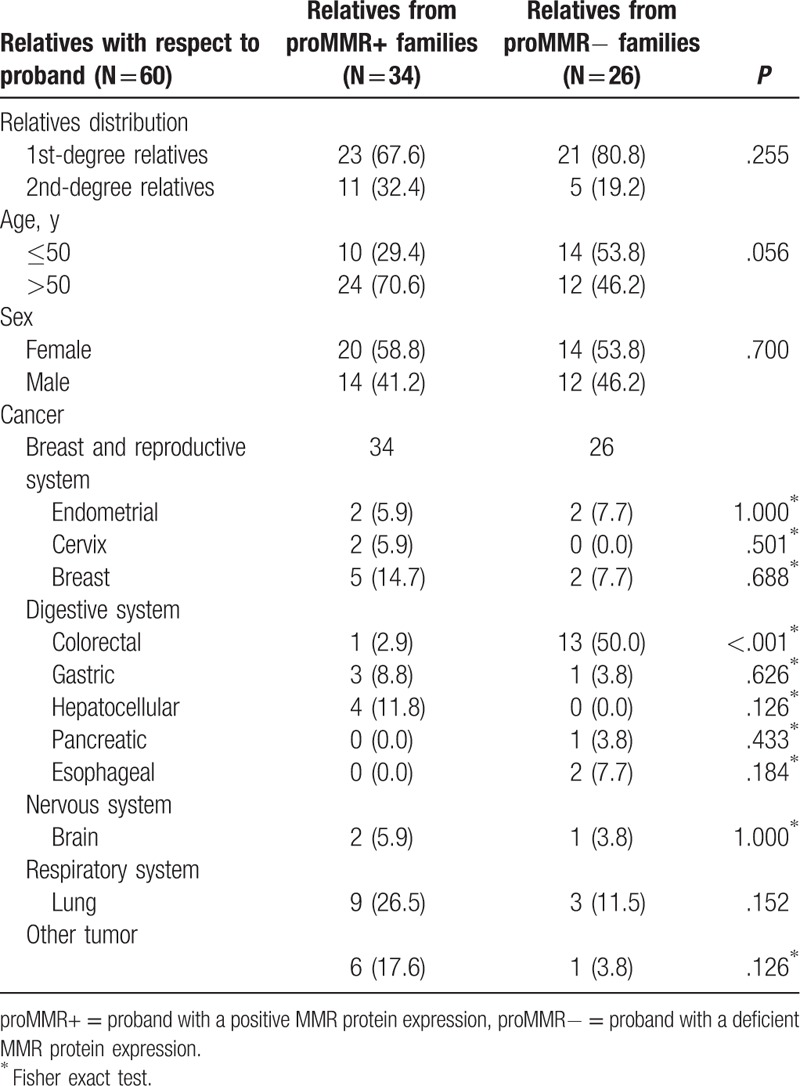
Family history of cancer in the PFH group.

Reproductive health history and the clinicopathology characteristics of patients are shown in Table 2. The Median (25%, 75%) age was 44 (38 and 46) years for the NFH group and 46 (41 and 49) years for the PFH group. The proportion of younger patients (age ≤ 40 years) was 40% (16/40) in the PFH group and 22.8% in the NFH group (*P* = .023). Twenty percent (8/40) of patients in the PFH group reported menarche at age ≤ 12 years, and this proportion was as low as 8.2% in the NFH group (*P* = .024). The proportion of obese was 9.5% in NFH group and 5.0% in PFH group, respectively, (*P* = .396). Regarding pathologic features, the majority of cases were endometroid histology (higher than 85%), and the proportion of lymph node metastasis was no more than 8% in either group. Most EC cases were diagnosed at an early stage (FIGO stage I) in both groups (83.60–92.5%). The rate of cervical involvement was 7.5% (3/40) in the PFH group and 22.8% in the NFH group (*P* < .05)

**Table 2:**
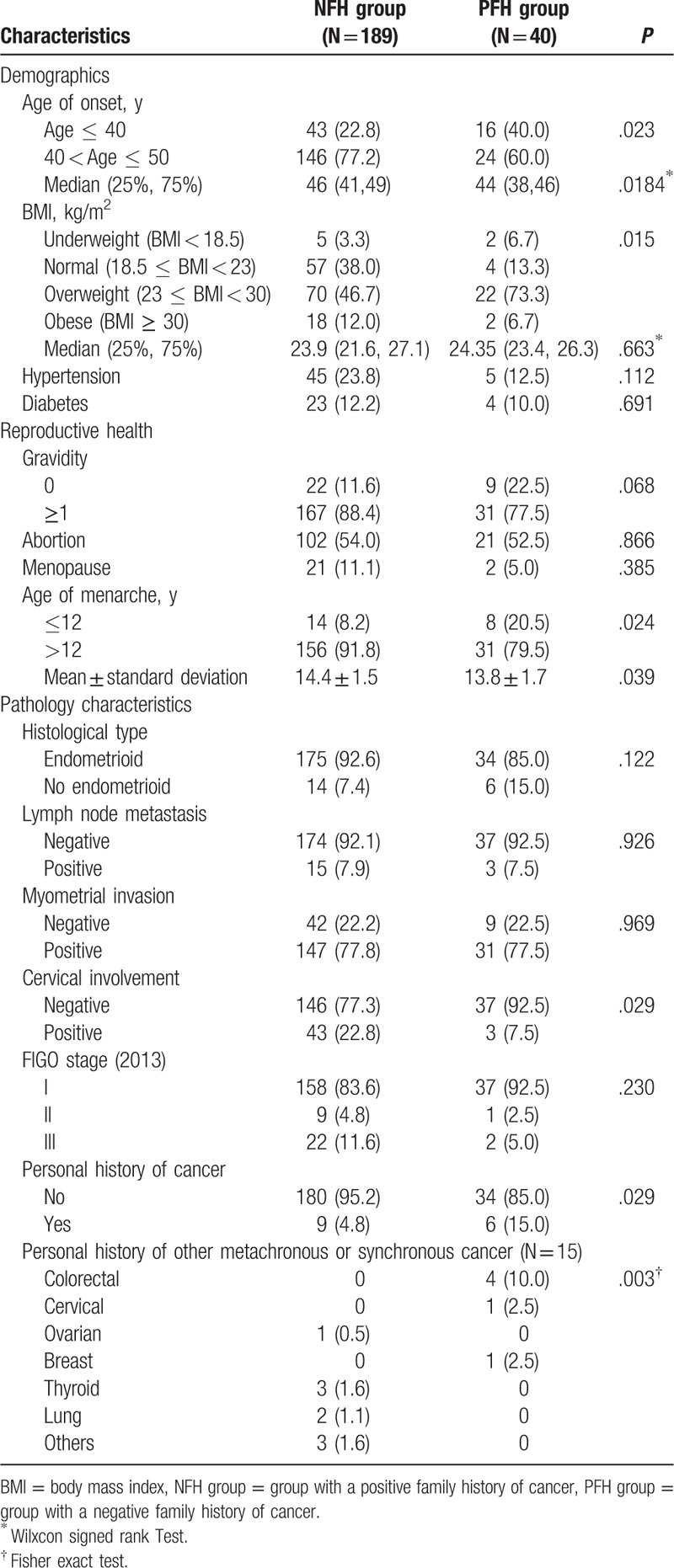
Demographic, reproductive health, and clinicopathology characteristics information of patients in the NFH and PFH groups.

Next, we assessed MMR protein expression in the 2 groups (Table 3). Of the 229 patients, 44/229 (19.2%) had deficiency in at least 1 MMR protein based on IHC analysis. Moreover, 14.3% (27/189) of patients had deficient MMR expression in the NFH group, while the proportion in the PFH group was 42.5% (17/40), which was nearly 3 times that in the NFH group (*P* < .001) (Table 3). In the NFH group, the rate of concurrent deficiency in MLH1 and PMS2 (4.2%) was similar to that in MSH2 and MSH6 (4.2% vs 3.2%). However, in the PFH group, concurrent deficiency in MSH2 and MSH6 (22.5%) was more frequent than that in MLH1 and PMS2 (7.5%) (*P* < .001).

**Table 3:**
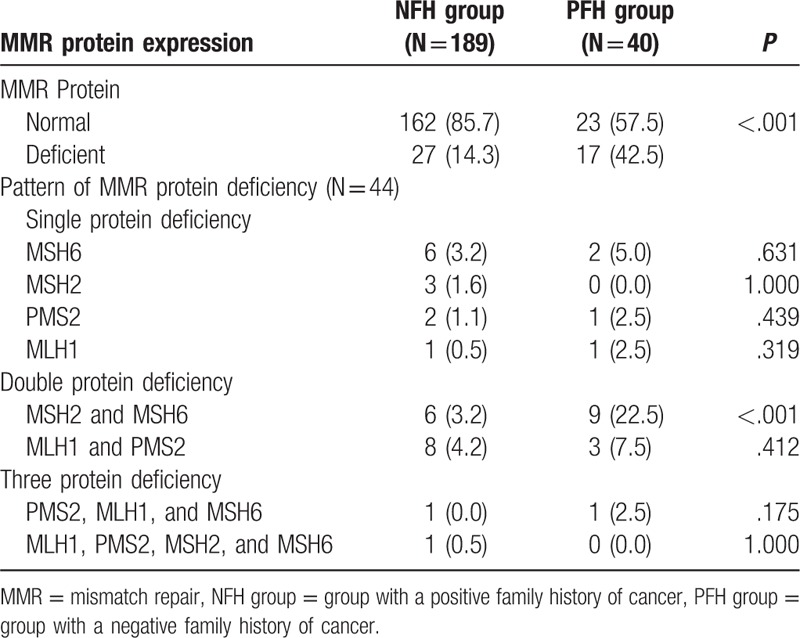
Pattern of MMR protein deficiency in the NFH and PFH groups.

The association between family history of cancer and clinicopathologic characteristics and MMR protein expression are shown in Table 4. Though person history of other cancer and cervical involvement were statistically significance differences between the PFH and NFH groups in the univariate statistics, no statistically significant difference in the adjusted ORs. However, cases with EC in the PFH group had a higher risk of younger age-of-onset of endometrial cancer (age ≤ 40) (adjusted OR = 2.21 with 95% confidence interval [95% CI]: 1.01–4.82) than cases in the NFH group in the stepwise multivariate logistic regression. In addition, women in the PFH group had a 4.81 times (95% CI: 2.14–8.83) risk of MMR protein deficiency after adjusting for related clinicopathologic variables in the multivariate logistic models.

**Table 4:**
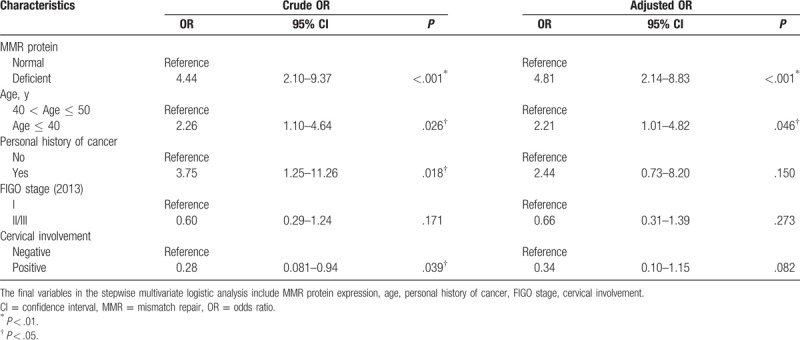
Relationship between family history (positive vs negative) and clinicopathologic characteristics.

## 4 Discussion

Understanding the influence of family history and age-of-onset of endometrial cancer will help to inform the clinical counseling and screening of high-risk LS families.^[19]^ This study proposed a universal IHC screening test of MMR protein and comprehensive evaluation of family history of cancer for the Chinese young (age ≤ 50) women with endometrial cancer. Our results indicate that women in the PFH group had a statistically significantly increased 2.21-fold (95% CI: 1.01–4.82) risk of younger age-of-onset (aged ≥ 40) of endometrial cancer than patients in NFH group. Young age-of-onset and family history of cancer, the clues of selecting patients for enhanced screening, may be influence by the same inherited factors. Whether only women with suggestive of LS or all women diagnosed with endometrial cancer should be screened for LS, continued to be debated. Universal screening of EC in individuals aged 50 years or younger has been suggested as a cost-effective strategy.^[8,20]^ However, Screening only patients under the age of 50 would miss at least 50% of LS cases.^[21]^ Screening cases aged < 70 years also would have failed to identify 12.5% of patients who had LS.^[22]^ The American College of Obstetrics and Gynecology and the Society of Gynecologic Oncology practice guidelines recommend that all women with endometrial cancer should undergo comprehensive clinical screening or molecular tumor-based testing.^[23]^ Screening based on family history and young age-of-onset alone may be inadequate in evaluating a patient for further testing for LS.

In this study, 40 (17.5%) out of 229 patients with EC had a family history of cancer. Colorectal, lung, endometrial, breast, and hepatocellular (58.6%) carcinoma were the main cancer types in FDRs and SDRs with cancer. The proportion of FDRs with cancer in proMMR− families was 13.1%, which was higher than that of relatives in proMMR+ families (*P* < .05). One of the most important reasons for these results might be the accumulation of MMR-related genetic factors. However, 34 relatives with cancer came from proMMR+ families, suggesting that the shared environmental factors between family members or gene–environmental interaction factors may play a role in the family history of cancer. A sufficiently detailed family history of cancer is a useful tool to allow the application of the LS criteria and further assessment.^[24]^ Thus, surveillance systems to capture sufficient family history information and risk-appropriate screening behaviors should be implemented for young women with EC in China.

The study showed the loss of MMR protein expression was reported in 44 (19.2%) patients with EC, which was within the range (19–38%) reported in other studies.^[16,25,26]^ This study did not aim to determine the methodologies to identify LS, but the loss of MMR protein expression was found to be associated with an increased positive family history of cancer (adjusted OR = 4.81, 95% CI: 2.14–8.83). Although the mechanism of loss of MMR protein expression in EC has not yet been fully confirmed, MMR protein expression can be affected by germline mutations or epigenetic modifications (such as promoter hypermethylation) of MLH1.^[27,28]^ The concurrent loss of MSH2 and MSH6 in the PFH group may suggest a risk for LS. However, the concurrent loss of MLH1 and PMS2, may be related to MLH1 and PMS2 gene mutations or MLH1 hypermethylation. Kimberly Resnick thought IHC evaluation of tumor specimens for MMR protein expression after single gene sequencing for patients with endometrial cancer is a cost-effective strategy for detecting LS.^[29]^ In China, restricted by the medical resources, IHC testing of MMR protein were usually concentrated in tertiary urban hospitals and largely performed according to the preferences of clinicians in China. With support from medical insurance providers, enhancing provider–patient knowledge of guide lines and encouraging young patients with EC to undergo IHC screening may be an effective strategy for overcoming barriers. Further research is needed to investigate screening strategies for LS in young women with EC in China.

Our study showed that a family history of cancer was not correlated with gravidity, abortion, hypertension, FIGO stage, or pathologic features. One of the possible explanations was that sample size in our study might have been insufficient to detect significance. Another explanation was the testing method was not sensitive enough to detect the difference between the PFH and NFH groups. However, nearly 80% of women in the PFH group and 58.7% of women in the NFH group had a BMI of ≥23 kg/m^2^ (overweight/obese). Traditionally young patients with EC with a family history of cancer may have a risk of being LS carriers. The increased proportion of overweight/obese patients in both the PFH and NFH groups indicates that LS risk does not fully explain the age-of-onset of EC. BMI may be yet another risk for young women to develop EC. Industrialization, fast-food diets and socioeconomic development have resulted in dramatic increases in BMI.^[30,31]^ The prevalence of overweight/obesity is 37.1% in urban residents, and 6.94% of EC cases have been attributed to overweight or obesity in China.^[30,32,33]^ Thus, reducing barriers to healthy lifestyles may be an urgent issue in China to prevent EC in women aged 50 years or younger.

### 4.1 Limitation

Strengths of the study included prospective data and extensive follow-up of MMR expression and family history of cancer. There are some limitations in the study. All data were obtained from one of the largest obstetrics and gynecology teaching hospitals in China over the last 2 years, which may not represent generalizable findings for China. However, the patients came from 5 provinces, which may have decreased the variance at the regional level. Another limitation was that the family history of cancer was self-reported by the patients. Challenges in communicating with family members may have led to recall bias in the collection of family history of cancer, but the pedigree from each proband was verified by their relatives during the study.

## 5 Conclusion

Patients with a positive family history of cancer had a 2.21-times increased risk of younger age-of-onset of endometrial cancer and 4.81-fold increased risk of MMR protein deficiency, which may be partly related to specific genetic or environmental factors or their interactions. Screening based on family history and young patient age alone may be inadequate in evaluating a patient for further testing for LS. Overweight/obesity is an urgent issue in patients with EC aged 50 years or younger. Colorectal, lung, endometrial, breast, and hepatocellular carcinoma accounted for approximately 58.5% of all cancer cases in the 2-generation pedigree. Surveillance of age-of-onset of endometrial cancer and family history of cancer, reduction of barriers to healthy lifestyles, and development of risk-appropriate LS screening tools, such as IHC methods, are needed for this subgroup of women in Shanghai and other developing cities in China.

## Acknowledgment

The authors thank Shanghai Municipal Science and Technology Committee for the funding support.

## Author contributions

Project administration: Weiwei Feng

Conception and design: Weiwei Feng, Jinming Yu

Collection and assembly of data: Xiao Tao, Yuan He, Nan Jia, Feifei Huang

Data analysis and interpretation: Yuan He, Jinming Yu, Yan Du

Manuscript writing, final approval of manuscript, accountable for all the work: All authors

**Conceptualization:** Weiwei Feng, Jinming Yu, Yan Du.

**Data curation:** Yuan He, Jinming Yu, Xiang Tao, Feifei Huang, Yan Du.

**Formal analysis:** Yuan He.

**Funding acquisition:** Weiwei Feng.

**Investigation:** Weiwei Feng, Yuan He, Feifei Huang, Nan Jia.

**Methodology:** Jinming Yu.

**Project administration:** Weiwei Feng, Yuan He.

**Resources:** Weiwei Feng, Xiang Tao.

**Software:** Yuan He.

**Visualization:** Jinming Yu.

**Writing – original draft:** Weiwei Feng, Yuan He, Jinming Yu.

## References

[1] Morice P Leary A Creutzberg C Endometrial cancer. Lancet 2016;387:1094–108. 26354523 10.1016/S0140-6736(15)00130-0

[2] Stewart BW Wild CP World Cancer Report 2014. France: The International Agency for Research on Cancer; 2014. 39432694

[3] Wei KR Chen WQ Zhang SW Epidemiology of uterine corpus cancer in some cancer registering areas of China from 2003–2007 [in Chinese]. Zhonghua Fu Chan Ke Zhi 2012;47:445–51. 22932112

[4] Zhang F Li J Zhou J A incidence trend analysis of cancer incidence in Minhang district of Shanghai from 2002 to 2013. Fudan Univ J Med Sci 2017;44:567–73.

[5] Chen W Zheng R Baade PD Cancer statistics in China, 2015. CA Cancer J Clin 2016;66:115–32. 26808342 10.3322/caac.21338

[6] Zhou JJ Fu ZX Wang YJ Trends of incidence and mortality of common gynecological malignant tumors among female residents in Luwan district of Shanghai. China Cancer 2016;25:854–9.

[7] Wei KR Liang ZH Ou ZX Epidemiology of female corpus uteri carcinoma in China. Pract Prev Med 2014;21:1150–3.

[8] Kwon JS Scott JL Gilks CB Testing women with endometrial cancer to detect Lynch syndrome. J Clin Oncol 2011;29:2247–52. 21537049 10.1200/JCO.2010.32.9979 PMC4874206

[9] Lynch HT Snyder CL Shaw TG Nature-milestones of Lynch syndrome: 1895-2015. Nat Rev Cancer 2015;15:181–94. 25673086 10.1038/nrc3878

[10] Win AK Reece JC Ryan S Family history and risk of endometrial cancer: a systematic review and meta-analysis. Obstet Gynecol 2015;125:89–98. 25560109 10.1097/AOG.0000000000000563

[11] Vasen HF Watson P Mecklin JP New clinical criteria for hereditary nonpolyposis colorectal cancer (HNPCC, Lynch syndrome) proposed by the International collaborative group on HNPCC. Gastroenterology 1999;116:1453–6. 10348829 10.1016/s0016-5085(99)70510-x

[12] Umar A Boland CR Terdiman JP Revised Bethesda guidelines for hereditary nonpolyposis colorectal cancer (Lynch syndrome) and microsatellite instability. J Natl Cancer Inst 2004;96:261–8. 14970275 10.1093/jnci/djh034 PMC2933058

[13] Dempsey KM Broaddus R You YN Is it all Lynch syndrome?: An assessment of family history in individuals with mismatch repair-deficient tumors. Genet Med 2015;17:476–84. 25341111 10.1038/gim.2014.131 PMC4936192

[14] Lucenteforte E Talamini R Montella M Family history of cancer and the risk of endometrial cancer. Eur J Cancer Prev 2009;18:95–9. 19337055 10.1097/CEJ.0b013e328305a0c9

[15] Cook LS Nelson HE Stidley CA Endometrial cancer and a family history of cancer. Gynecol Oncol 2013;130:334–9. 23632205 10.1016/j.ygyno.2013.04.053 PMC4052607

[16] McMeekin DS Tritchler DL Cohn DE Clinicopathologic significance of mismatch repair defects in endometrial cancer: an NRG oncology/gynecologic oncology group study. J Clin Oncol 2016;34:3062–8. 27325856 10.1200/JCO.2016.67.8722 PMC5012715

[17] Kastrinos F Steyerberg EW Mercado R The PREMM(1,2,6) model predicts risk of MLH1, MSH2, and MSH6 germline mutations based on cancer history. Gastroenterology 2011;140:73–81. 20727894 10.1053/j.gastro.2010.08.021 PMC3125673

[18] WHO Expert Consultation. Appropriate body-mass index for Asian populations and its implications for policy and intervention strategies. Lancet 2004;363:157–63. 14726171 10.1016/S0140-6736(03)15268-3

[19] Bruegl AS Ring KL Daniels M Clinical challenges associated with universal screening for Lynch syndrome-associated endometrial cancer. Cancer Prev Res 2017;10:108–15. 10.1158/1940-6207.CAPR-16-0219 PMC5292079 27965287

[20] Ferguson SE Aronson M Pollett A Performance characteristics of screening strategies for Lynch syndrome in unselected women with newly diagnosed endometrial cancer who have undergone universal germline mutation testing. Cancer 2014;120:3932–9. 25081409 10.1002/cncr.28933

[21] de la Chapelle A Palomaki G Hampel H Identifying Lynch syndrome. Int J Cancer 2009;125:1492–3. 19536819 10.1002/ijc.24491 PMC2771416

[22] Adar T Rodgers LH Shannon KM Universal screening of both endometrial and colon cancers increases the detection of Lynch syndrome. Cancer 2018;124:3145–53. 29750335 10.1002/cncr.31534

[23] Committee on Practice B-G, Society of Gynecologic Oncology. ACOG Practice Bulletin No 147: Lynch syndrome. Obstet Gynecol 2014;124:1042–54. 25437740 10.1097/01.AOG.0000456325.50739.72

[24] Eiriksson L Aronson M Clarke B Performance characteristics of a brief family history questionnaire to screen for Lynch syndrome in women with newly diagnosed endometrial cancer. Gynecol Oncol 2015;136:311–6. 25529831 10.1016/j.ygyno.2014.12.023

[25] Steinhagen E Shia J Markowitz AJ Systematic immunohistochemistry screening for Lynch syndrome in early age-of-onset colorectal cancer patients undergoing surgical resection. J Am Coll Surg 2012;214:61–7. 22192923 10.1016/j.jamcollsurg.2011.10.004

[26] Mills AM Sloan EA Thomas M Clinicopathologic comparison of Lynch syndrome-associated and “Lynch-like” endometrial carcinomas identified on Universal screening using mismatch repair protein immunohistochemistry. Am J Surg Pathol 2016;40:155–65. 26523542 10.1097/PAS.0000000000000544

[27] Hecht JL Mutter GL Molecular and pathologic aspects of endometrial carcinogenesis. J Clin Oncol 2006;24:4783–91. 17028294 10.1200/JCO.2006.06.7173

[28] Stelloo E Jansen AML Osse EM Practical guidance for mismatch repair-deficiency testing in endometrial cancer. Ann Oncol 2017;28:96–102. 27742654 10.1093/annonc/mdw542

[29] Resnick K Straughn M Jr Backes F Lynch syndrome screening strategies among newly diagnosed endometrial cancer patients. Obstetrics Gynecology 2009;114:530–6. 19701031 10.1097/AOG.0b013e3181b11ecc

[30] Gao Y Dai X Chen L Body mass index is positively associated with endometrial cancer in Chinese women, especially prior to menopause. J Cancer 2016;7:1169–73. 27326261 10.7150/jca.15037 PMC4911885

[31] Huang Z Beeghly-Fadiel AC Zheng Y Abstract 4140: Secular trends in incidence and mortality of female cancers in Shanghai, China (1973-2009). Cancer Res 2014;74:4140.

[32] Hu L Huang X You C Prevalence of overweight, obesity, abdominal obesity and obesity-related risk factors in southern China. PLoS One 2017;12:e0183934. 28910301 10.1371/journal.pone.0183934 PMC5598943

[33] Gao J Yang G Wen W Impact of known risk factors on endometrial cancer burden in Chinese women. Eur J Cancer Prev 2016;25:329–34. 26075656 10.1097/CEJ.0000000000000178 PMC4676733

